# Supplementary material for: Bias in the reporting of sex and age in biomedical research on mouse models
Source: eLife. 2016 Mar 3;5:e13615. doi: 10.7554/eLife.13615 (PMC4821800; doi:10.7554/eLife.13615)
Supplement: Figure 1—source data 1. — DOI: http://dx.doi.org/10.7554/eLife.13615.004 [file elife-13615-fig1-data1.docx]

**Figure 1–source data 1.** PubMed search terms used for each disease group and their approaches.

| **Groups** | **Search terms** | **Number of articles** |
| --- | --- | --- |
| **Main corpus** | ("Mice"[Mesh] AND (mouse[ti] OR mice[ti])) AND (Journal Article[ptyp] NOT Review[ptyp] AND ("1994/01/01"[PDAT] : "2014/12/31"[PDAT]) AND "animals"[MeSH Terms:noexp] AND English[lang]) AND pubmed pmc[sb] | 15,311 |
| **Subsets** | ("Mice"[Mesh] AND (mouse[ti] OR mice[ti])) AND (Journal Article[ptyp] NOT Review[ptyp] AND ("2001/01/01"[PDAT] : "2014/12/31"[PDAT]) AND "animals"[MeSH Terms:noexp] AND English[lang]) AND pubmed pmc[sb] | 14,225 |
| **Cardiovascular diseases** | AND "Cardiovascular Diseases"[Mesh] | 873 |
| Genetics | AND "Cardiovascular Diseases/genetics"[Mesh] | 293 |
| Immunology | AND "Cardiovascular Diseases/immunology"[Mesh] | 58 |
| Physiopathology | AND "Cardiovascular Diseases/physiopathology"[Mesh] | 231 |
| Therapy | AND "Cardiovascular Diseases/therapy"[Mesh] | 320 |
| Myocardial Ischemia | AND "Myocardial Ischemia"[Mesh] | 94 |
| **Cancer** | AND "Neoplasms"[Mesh] | 1,523 |
| Genetics | AND "Neoplasms/genetics"[Mesh] | 604 |
| Immunology | AND "Neoplasms/immunology"[Mesh] | 179 |
| Physiopathology | AND "Neoplasms/physiopathology"[Mesh] | 49 |
| Therapy | AND "Neoplasms/therapy"[Mesh] | 540 |
| Melanoma | AND "Melanoma"[Mesh] | 98 |
| **Diabetes Mellitus** | AND "Diabetes Mellitus"[Mesh] | 611 |
| Genetics | AND "Diabetes Mellitus/genetics"[Mesh] | 183 |
| Immunology | AND "Diabetes Mellitus/immunology"[Mesh] | 108 |
| Physiopathology | AND "Diabetes Mellitus/physiopathology"[Mesh] | 112 |
| Therapy | AND "Diabetes Mellitus/therapy"[Mesh] | 243 |
| Diabetes type 2 | AND "Diabetes Mellitus, Type 2"[Mesh] | 149 |
| **Lung diseases** | AND "Lung Diseases"[Mesh] | 602 |
| Genetics | AND "Lung Diseases/genetics"[Mesh] | 153 |
| Immunology | AND "Lung Diseases/immunology"[Mesh] | 162 |
| Physiopathology | AND "Lung Diseases/physiopathology"[Mesh] | 76 |
| Therapy | AND "Lung Diseases/therapy”[Mesh] | 191 |
| Diabetes type 2 | AND "Pulmonary Disease, Chronic Obstructive”[Mesh] | 37 |
| **Neurological disorders** | AND "Nervous System Diseases"[Mesh] | 2,137 |
| Genetics | AND "Nervous System Diseases/genetics"[Mesh] | 899 |
| Immunology | AND "Nervous System Diseases/immunology"[Mesh] | 182 |
| Physiopathology | AND "Nervous System Diseases/physiopathology"[Mesh] | 556 |
| Therapy | AND "Nervous System Diseases/therapy"[Mesh] | 609 |
| Alzheimer | AND "Alzheimer Disease”[Mesh] | 273 |
| **Infectious diseases** | AND (“microbiology” [Subheading] OR “virology” [Subheading] OR “Parasitology” [Subheading]) | 1,269 |
| Physiopathology | AND ("microbiology" [Subheading] OR "virology" [Subheading] OR "Parasitology" [Subheading]) AND "physiopathology" [Subheading] | 67 |
| Genetics | AND ("microbiology" [Subheading] OR "virology" [Subheading] OR "Parasitology" [Subheading]) AND "genetics" [Subheading] | 640 |
| Immunology | AND ("microbiology" [Subheading] OR "virology" [Subheading] OR "Parasitology" [Subheading]) AND "immunology" [Subheading] | 662 |
| Therapy | AND ("microbiology" [Subheading] OR "virology" [Subheading] OR "Parasitology" [Subheading]) AND "therapy" [Subheading] | 370 |
| Tuberculosis | AND ("microbiology" [Subheading] NOT "virology" [Subheading] NOT "Parasitology" [Subheading]) AND "Tuberculosis"[Mesh] | 39 |
| HIV | AND ("virology" [Subheading] NOT "Parasitology" [Subheading]) AND "HIV"[Mesh] | 62 |
| Malaria | AND ("Parasitology" [Subheading]) AND "Malaria"[Mesh] | 39 |

Terms were chosen to cover both PubMed MeSH (Medical Subject Headings) and related strings to ensure that articles would still be captured even if they lacked correct subject heading annotations.
