# Supplementary material for: Bias in the reporting of sex and age in biomedical research on mouse models
Source: eLife. 2016 Mar 3;5:e13615. doi: 10.7554/eLife.13615 (PMC4821800; doi:10.7554/eLife.13615)
Supplement: Figure 1—source data 2. — DOI: http://dx.doi.org/10.7554/eLife.13615.005 [file elife-13615-fig1-data2.docx]

**Figure 1–source data 2.** Example rules for identification of sex and age.

| **Characteristics** | **Rules** | **Phrase** | | | | | | | | | | | | |
| --- | --- | --- | --- | --- | --- | --- | --- | --- | --- | --- | --- | --- | --- | --- |
| Sex | Abstract rule | Gender (adjective) | | | | | Mice (noun phrase) | | | | | | | |
|  | Rule example | ({Token.string==~"(?i)male"}) | | | | | {Token.string==~"(?i)mice"} | | | | | | | |
|  | Male mice 6-8-wk-old | Male | | | | | mice | | | | | | | |
|  |  | | | | | | | | | | | | | |
|  | Abstract rule | Mice (noun phrase) | | Preposition | | | Conjunction | | | | Gender | | | |
|  | Rule example | {Token.string==~"(?i)mice"} | | {Token.string ==~"(?i)of"} | | | ({Token.string ==~"(?i)either"} | | | | {Token.string ==~"(?i)sex"}) | | | |
|  | Mice of either sex were used | Mice | | of | | | either | | | | Sex | | | |
|  | | | | | | | | | | | | | | |
| Age | Abstract rule | Mice (noun phrase) | | | Verb | | | Numeric dictionary | Any token | | | Numeric dictionary | | Age (noun phrase) |
|  | Rule example | {Token.string==~"(?i)mice"} | | | {Token.string=="were"} | | | ((numbers) | {Token}[0,1] | | | (numbers)? | | (age)) |
|  | Mice were 4 wk old | mice | | | were | | | 4 |  | | |  | | wk old |
|  |  | | | | | | | | | | | | | |
|  | Abstract rule | Numeric dictionary | Any token | | | Numeric dictionary | | Age (noun phrase) | | Any token | | | Mice (noun phrase) | |
|  | Rule example | ((numbers) | {Token}[0,1] | | | (numbers)? | | (age)) | | {Token}[0,2] | | | {Token.string==~"(?i)mice"} | |
|  | Generally 3-4 months old healthy adult mice | 3 | - | | | 4 | | months old | | healthy adult | | | mice | |

Examples show both an “abstract” description of the rule and the applied GATE notation. Rule components in highlighted text are the extracted (target) text that denote the mention of interest; the rest of the rule (if any) specifies the context. The rules use explicit matching of tokens (e.g., {Token.string ==~ "(?i)male"} matches the string ‘male’) and vocabularies that contain mentions of specific dictionaries. For example, (age) matches variations of the expressed age (e.g., ‘wk old’, ‘months old’) and (numbers) contains multiple numbers in both arithmetic and lexical forms. The ‘?’ at the end of certain rule components suggests ‘if any’, whereas {Token}[0,1] matches up to the given number of tokens, if any.
