# Supplementary material for: Bias in the reporting of sex and age in biomedical research on mouse models
Source: eLife. 2016 Mar 3;5:e13615. doi: 10.7554/eLife.13615 (PMC4821800; doi:10.7554/eLife.13615)
Supplement: Supplementary file 2. — (B) Set of articles for finding the location of the mention of the sex and age of the mice. (C) Set of articles for enhancing the performance of the text-mining rules. (D) Set of articles for evaluating the text-mining system. DOI: http://dx.doi.org/10.7554/eLife.13615.013 [file elife-13615-supp2.docx]

**Supplementary File 2A**

**SET OF ARTICLES FOR CREATING THE TEXT-MINING RULES**

The documents were manually extracted from PubMed using the query "Mice"[Mesh] AND (mouse[ti] OR mice[ti]) AND Journal Article[ptyp] AND English[lang].

**PMID Journal Year**

15642986 Clin Diagn Lab Immunol 2005

19254734 Physiol Behav 2009

21185930 J Ethnopharmacol 2011

21190827 J Nutr Biochem 2011

21193983 Psychopharmacology (Berl) 2011

21199659 J Immunol Methods 2011

21218482 J Sci Food Agric 2011

24015257 PLoS One 2013

24534203 Cancer Lett 2014

24646876 Immunobiology 2014

24736856 J Antibiot (Tokyo) 2014

24776490 Behav Pharmacol 2014

24871354 J Nat Prod 2014

24887420 PLoS One 2014

25069986 Infect Immun 2014

25201301 Br J Nutr 2014

25217696 Blood 2014

25218594 Cancer Lett 2014

25224570 Cancer Lett 2014

25231351 Am J Physiol Regul Integr Comp Physiol 2014

25234596 Biochem Biophys Res Commun 2014

25245810 Infect Immun 2014

25246326 Exp Parasitol 2014

25261995 Nat Med 2014

25267834 Infect Immun 2014

25268558 J Toxicol Environ Health A 2014

25273880 Am J Physiol Cell Physiol 2014

25280587 BMC Complement Altern Med 2014

25282357 Nat Med 2014

25287930 Infect Immun 2014

25288643 J Med Microbiol 2014

25288806 J Biol Chem 2014

25303897 Exp Mol Pathol 2014

25308446 Metabolism 2014

25318387 BMC Complement Altern Med 2014

25320354 Am J Physiol Renal Physiol 2014

25355549 BMC Complement Altern Med 2014

25367573 Immunity 2014

25283970 Prostate 2015

25347995 J Pharmacol Exp Ther 2015

**Supplementary File 2B**

**SET OF ARTICLES FOR FINDING THE LOCATION OF THE MENTION OF THE SEX AND AGE OF THE MICE**

The documents were randomly extracted from our corpus by using the “=RANDBETWEEN()” function in Microsoft Office Excel for Windows version 2013, and manually inspected in order to determine in which part of the article the sex and age of the mice were mentioned.

**PMID Journal Year**

8976197 J Exp Med 1996

8976195 J Exp Med 1996

8976192 J Exp Med 1996

9049243 J Cell Biol 1997

9034144 J Exp Med 1997

9008713 J Cell Biol 1997

9808781 Dev Biol 1998

16172261 J Exp Med 2005

17900358 BMC Neurosci 2007

17683525 BMC Neurosci 2007

17592641 J Transl Med 2007

19020657 PLoS One 2008

18688274 PLoS Pathog 2008

18568131 Mol Vis 2008

19765281 Arthritis Res Ther 2009

20405019 PLoS One 2010

20368974 PLoS One 2010

20098691 PLoS One 2010

23272179 PLoS One 2012

23237483 BMC Immunol 2012

22802958 PLoS One 2012

21765465 Oncogene 2012

24278473 PLoS One 2013

24212843 Clinics (Sao Paulo) 2013

24194903 PLoS One 2013

24147098 PLoS One 2013

24098534 PLoS One 2013

23966857 PLoS Pathog 2013

23903059 Exp Anim 2013

23762356 PLoS One 2013

23667681 PLoS One 2013

23613811 PLoS One 2013

23516562 PLoS One 2013

23451234 PLoS One 2013

23326190 Int J Nanomedicine 2013

23321513 Br J Cancer 2013

23302418 BMC Neurosci 2013

23286586 J Biomed Sci 2013

24995344 J Immunol Res 2014

24455991 J Cell Mol Med 2014

**Supplementary File 2C**

**SET OF ARTICLES FOR ENHANCING THE PERFORMANCE OF THE TEXT-MINING RULES**

The documents were randomly extracted from our corpora by using the “=RANDBETWEEN()” function in Microsoft Office Excel for Windows version 2013. Five documents were used for each year from 2001 to 2014.

**PMID Journal Year**

11305942 Genome Biol 2001

11304550 J Exp Med 2001

11748281 J Exp Med 2001

11785668 Dev Immunol 2001

11737881 BMC Complement Altern Med 2001

12021255 J Cell Biol 2002

12401133 BMC Cell Biol 2002

12163565 J Exp Med 2002

12198088 J Gen Physiol 2002

11956298 J Exp Med 2002

14623911 J Exp Med 2003

12925704 J Cell Biol 2003

12860970 J Cell Biol 2003

12932298 Reprod Biol Endocrinol 2003

12771178 J Exp Med 2003

15483348 J Korean Med Sci 2004

14728723 BMC Neurosci 2004

15302899 J Exp Med 2004

15534693 PLoS Biol 2004

15154615 Clin Dev Immunol 2004

16250671 PLoS Med 2005

15998448 Genome Biol 2005

16293190 BMC Infect Dis 2005

16033648 BMC Dev Biol 2005

16079067 Environ Health Perspect 2005

16571105 BMC Genet 2006

16563162 Mol Cancer 2006

16502487 Yonsei Med J 2006

17069643 BMC Gastroenterol 2006

17069661 Virol J 2006

17406675 PLoS ONE 2007

17683579 BMC Cancer 2007

17266762 Genome Biol 2007

17220887 Nat Neurosci 2007

17605779 BMC Dev Biol 2007

18371231 BMC Genomics 2008

18716442 J Vet Sci 2008

18547429 Lipids Health Dis 2008

18307760 Respir Res 2008

18789160 BMC Cell Biol 2008

19750022 Toxicol Mech Methods 2009

19557135 PLoS One 2009

19129917 PLoS One 2009

19296832 BMC Microbiol 2009

19221395 J Exp Med 2009

20525357 BMC Biol 2010

20041326 Cancer Chemother Pharmacol 2010

20689830 PLoS One 2010

20796285 BMC Neurosci 2010

21171988 BMC Genomics 2010

21818344 PLoS One 2011

21799730 PLoS One 2011

21412423 PLoS One 2011

22163031 PLoS One 2011

21439091 Malar J 2011

21954065 Dis Model Mech 2012

22235288 PLoS One 2012

22275470 Dis Model Mech 2012

23087911 Front Cell Infect Microbiol 2012

22859963 PLoS One 2012

23967191 PLoS One 2013

23536174 Sci Rep 2013

23451234 PLoS One 2013

24317954 Oncotarget 2013

23519026 Dis Model Mech 2013

24466007 PLoS One 2014

25077564 BMC Genomics 2014

24924430 Dis Model Mech 2014

24638941 Int J Mol Med 2014

24559113 BMC Complement Altern Med 2014

**Supplementary File 2D**

**SET OF ARTICLES FOR EVALUATING THE TEXT-MINING SYSTEM**

The documents were randomly extracted from our corpus by using the “=RANDBETWEEN()” function in Microsoft Office Excel for Windows version 2013, and manually double-annotated for both the age and the sex by two biomedical experts.

**PMID Journal Year**

8145050 J Exp Med 1994

7744960 J Cell Biol 1995

8924761 Dev Immunol 1995

8879219 J Exp Med 1996

9064345 J Exp Med 1996

10880524 J Exp Med 2000

11532190 BMC Cell Biol 2001

16800892 BMC Biotechnol 2006

18280460 Biochem Pharmacol 2008

19127268 Br J Cancer 2009

19255868 Biogerontology 2009

20041218 PLoS Genet 2009

19850720 Nucleic Acids Res 2010

19920212 Physiol Genomics 2010

20084100 PLoS Genet 2010

20107508 PLoS ONE 2010

20167811 Neuro Oncol 2010

20169060 PLoS ONE 2010

20405007 PLoS ONE 2010

20686609 PLoS ONE 2010

20532624 Transgenic Res 2011

21464968 PLoS ONE 2011

21492450 BMC Neurosci 2011

22428884 J Environ Sci Health B 2012

22520439 J Neuroinflammation 2012

22532835 PLoS ONE 2012

22547652 J Exp Med 2012

22675511 PLoS ONE 2012

22892315 Mol Brain 2012

22906987 Lab Invest 2012

22952733 PLoS ONE 2012

23049968 PLoS ONE 2012

23194061 Reprod Biol Endocrinol 2012

23233794 Mol Vis 2012

23316291 J Am Heart Assoc 2012

23341968 PLoS ONE 2013

23935987 PLoS ONE 2013

23936125 PLoS ONE 2013

23942071 Br J Cancer 2013

23991183 PLoS ONE 2013

24386094 PLoS ONE 2013

24459328 Mediators Inflamm 2013

24273196 J Lipid Res 2014

24361736 Neuroscience 2014

24493738 Nucleic Acids Res 2014

24500039 Med Sci Monit Basic Res 2014

24621297 Aging Cell 2014

24833816 Mediators Inflamm 2014

24877142 Biomed Res Int 2014

25092975 Int J Nanomedicine 2014
