# Supplementary material for: Bias in the reporting of sex and age in biomedical research on mouse models
Source: eLife. 2016 Mar 3;5:e13615. doi: 10.7554/eLife.13615 (PMC4821800; doi:10.7554/eLife.13615)
Supplement: Supplementary file 3. — DOI: http://dx.doi.org/10.7554/eLife.13615.014 [file elife-13615-supp3.docx]

**Supplementary file 3**

**RULES USED TO IDENTIFY THE SEX AND AGE OF EXPERIMENTAL MOUSE MODELS**

Our rules were created and applied via GATE –General Architecture for Text Engineering; an open source free software enabling the design and implementation of information extraction systems in unstructured text with the crafted rules following its notation.

**Rules for the identification of sex**

(
{Token.string ==~ "(?i)male"}
|
{Token.string ==~ "(?i)female"}
|
{Token.string ==~ "(?i)males"}
|
{Token.string ==~ "(?i)females"}
|
({Token.string ==~ "(?i)female"}{Token.string == "/"}{Token.string ==~ "(?i)male"})
|
({Token.string ==~ "(?i)male"}{Token.string == "/"}{Token.string ==~ "(?i)female"})
|
({Token.string ==~ "(?i)male"}{Token.string ==~ "(?i)and"}{Token.string ==~ "(?i)female"})
|
({Token.string ==~ "(?i)female"}{Token.string ==~ "(?i)and"}{Token.string ==~ "(?i)male"})
|
(

({Token.string ==~"(?i)age"}|{Token.string ==~"(?i)sex"})
({Token.string == "-"})?
{Token.string ==~"(?i)and"}
({Token.string ==~"(?i)sex"}|{Token.string ==~"(?i)age"})
({Token.string == "-"})?
{Token.string ==~"(?i)matched"}
)
|
(
 ({Token.string ==~"(?i)age-"}|{Token.string ==~"(?i)sex-"})
{Token.string ==~"(?i)and"}
({Token.string ==~"(?i)sex-"}|{Token.string ==~"(?i)age-"})
{Token.string ==~"(?i)matched"}
)
|
(
({Token.string ==~"(?i)age-"}|{Token.string ==~"(?i)sex-"})
{Token.string ==~"(?i)and"}
({Token.string ==~"(?i)sex-matched"}|{Token.string ==~"(?i)age-matched"})
)
|
(
{Token.string ==~"(?i)mice"}
{Token.string ==~"(?i)of"}
({Token.string ==~"(?i)both"}|{Token.string==~"(?i)either"})
({Token.string ==~"(?i)sexes"}| {Token.string ==~"(?i)gender"})
)

**Frozen lexical expression used as anchors inside the rules for the identification of age**

Macro: gender
(
{Token.string ==~"(?i)male"}|{Token.string ==~"(?i)female"}|{Token.string ==~"(?i)males"}|{Token.string ==~"(?i)females"}
)

Macro: weeks
(
{Token.string ==~"(?i)mts"}
|
{Token.string ==~"(?i)months"}
|
{Token.string ==~"(?i)days"}
|
{Token.string ==~"(?i)week"}
|
{Token.string ==~"(?i)wk"}
|
{Token.string ==~"(?i)weeks"}
|
{Token.string ==~"(?i)wks"}
|
{Token.string ==~"(?i)month-old"}
|
{Token.string ==~"(?i)months-old"}
|
{Token.string ==~"(?i)mts-old"}
|
{Token.string ==~"(?i)week-old"}
|
{Token.string ==~"(?i)weeks-old"}
|
{Token.string ==~"(?i)wks-old"}
|
{Token.string ==~"(?i)wk-old"}
|
{Token.string ==~"(?i)day-old"}
|
{Token.string ==~"(?i)days-old"}
|
{Token.string ==~"(?i)months"}{Token.string =="–"}{Token.string ==~"(?i)old"}
|
{Token.string ==~"(?i)month"}{Token.string =="–"}{Token.string ==~"(?i)old"}
|
{Token.string ==~"(?i)mts"}{Token.string =="–"}{Token.string ==~"(?i)old"}
|
{Token.string ==~"(?i)week"}{Token.string =="–"}{Token.string ==~"(?i)old"}
|
{Token.string ==~"(?i)wk"}{Token.string =="–"}{Token.string ==~"(?i)old"}
|
{Token.string ==~"(?i)weeks"}{Token.string =="–"}{Token.string ==~"(?i)old"}
|
{Token.string ==~"(?i)wks"}{Token.string =="–"}{Token.string ==~"(?i)old"}
|
{Token.string ==~"(?i)days"}{Token.string =="–"}{Token.string ==~"(?i)old"}
|
{Token.string ==~"(?i)day"}{Token.string =="–"}{Token.string ==~"(?i)old"}
|
{Token.string ==~"(?i)month"}{Token.string ==~"(?i)old"}
|
{Token.string ==~"(?i)mts"}{Token.string ==~"(?i)old"}
|
{Token.string ==~"(?i)months"}{Token.string ==~"(?i)old"}
|
{Token.string ==~"(?i)wks"}{Token.string ==~"(?i)old"}
|
{Token.string ==~"(?i)weeks"}{Token.string ==~"(?i)old"}
|
{Token.string ==~"(?i)wk"}{Token.string ==~"(?i)old"}
|
{Token.string ==~"(?i)d"}{Token.string ==~"(?i)old"}
|
{Token.string ==~"(?i)week"}{Token.string ==~"(?i)old"}
|
{Token.string ==~"(?i)weeks"}{Token.string ==~"(?i)of"}{Token.string ==~"(?i)age"}
|
{Token.string ==~"(?i)wks"}{Token.string ==~"(?i)of"}{Token.string ==~"(?i)age"}
|
{Token.string ==~"(?i)week"}{Token.string ==~"(?i)of"}{Token.string ==~"(?i)age"}
|
{Token.string ==~"(?i)wk"}{Token.string ==~"(?i)of"}{Token.string ==~"(?i)age"}
|
{Token.string ==~"(?i)month"}{Token.string ==~"(?i)of"}{Token.string ==~"(?i)age"}
|
{Token.string ==~"(?i)months"}{Token.string ==~"(?i)of"}{Token.string ==~"(?i)age"}
|
{Token.string ==~"(?i)mts"}{Token.string ==~"(?i)of"}{Token.string ==~"(?i)age"}
|
{Token.string ==~"(?i)days"}{Token.string ==~"(?i)of"}{Token.string ==~"(?i)age"}
)

Macro: whole_string_age
(
{Token.string ==~"(?i)five-week-old"} |{Token.string ==~"(?i)six-week-old"}|{Token.string ==~"(?i)five-weeks-old"}|{Token.string ==~"(?i)six-weeks-old"}|{Token.string ==~"(?i)two-week-old"}|{Token.string ==~"(?i)three-week-old"}|{Token.string ==~"(?i)seven-week-old"}|{Token.string ==~"(?i)eight-week-old"}|{Token.string ==~"(?i)nine-week-old"}|{Token.string ==~"(?i)ten-week-old"}|{Token.string ==~"(?i)five-week"} |{Token.string ==~"(?i)six-week"}|{Token.string ==~"(?i)five-weeks"}|{Token.string ==~"(?i)six-weeks"}|{Token.string ==~"(?i)two-week"}|{Token.string ==~"(?i)three-week"}|{Token.string ==~"(?i)seven-week"}| {Token.string ==~"(?i)eight-week"}|{Token.string ==~"(?i)nine-week"}|{Token.string ==~"(?i)ten-week"}
)

Macro: wholte_string_age2
(
{Token.string ==~"(?i)five-"} |{Token.string ==~"(?i)six-"}|{Token.string ==~"(?i)two-"}|{Token.string ==~"(?i)three-"}|{Token.string ==~"(?i)seven-"}| {Token.string ==~"(?i)eight-"}| {Token.string ==~"(?i)nine-"}| {Token.string ==~"(?i)ten-"}
)

Macro: numbers
(
{Token.string ==~"[0-9]"}|{Token.string==~"[0-9]+"}|{Token.string ==~"(?i)one"}|{Token.string ==~"(?i)two"}|{Token.string ==~"(?i)three"}|{Token.string ==~"(?i)four"}|{Token.string ==~"(?i)five"}|{Token.string ==~"(?i)six"}|{Token.string ==~"(?i)seven"}|{Token.string ==~"(?i)eight"}|{Token.string ==~"(?i)nine"}|{Token.string ==~"(?i)ten"}|{Token.string ==~"(?i)eleven"}|{Token.string ==~"(?i)twelve"}|{Token.string ==~"(?i)thirteen"}|{Token.string ==~"(?i)fourteen"}
)

Macro: link
(
{Token.string ==~"(?i)to"}|{Token.string =="-"}|{Token.string =="–"}
)

**Rules for the identification of age**

(
({Token.string==~"(?i)embryos"}):age
)
|
(
{Token.string==~"(?i)mice"}
({Token})[0,2]
{Token.string==~"(?i)age"}
{Token.string==~"(?i)of"}
((numbers)
({Token})[0,1]
(numbers)?
(weeks)):age
)
|
(
{Token.string==~"(?i)mice"}
({Token})[0,1]
({Token.string==~"(?i)aged"})?
((numbers)
({Token})[0,1]
(numbers)?
(weeks)):age
)
|
(
{Token.string ==~"(?i)mice"}
{Token.string ==~"(?i)were"}
{Token.string ==~"(?i)used"}
{Token.string ==~"(?i)for"}
{Token.string ==~"(?i)experiments"}
{Token.string ==~"(?i)at"}
((numbers)
({Token})[0,1]
(numbers)?
(weeks)):age
)
|
(
{Token.string ==~"(?i)mice"}
{Token.string ==~"(?i)aged"}
{Token.string ==~"(?i)between"}
((numbers)
({Token})[0,1]
(numbers)?
(weeks)):age
)
|
(
{Token.string ==~"(?i)mice"}
{Token.string=="("}
{Token.string ==~"(?i)average"}
{Token.string ==~"(?i)age"}
((numbers)
({Token})[0,1]
(numbers)?
(weeks)):age
)
|
(
{Token.string ==~"(?i)mice"}
{Token.string ==~"(?i)were"}
{Token.string ==~"(?i)used"}
{Token.string ==~"(?i)before"}
((numbers)
({Token})[0,1]
(numbers)?
(weeks)):age
)
|
(
((numbers)
({Token})[0,1]
(numbers)?
(weeks)):age
({Token})[0,2]
{Token.string==~"(?i)mice"}
)
|
(
((numbers)
({Token})[0,1]
(numbers)?
(weeks)):age
({Token})[0,1]
{Token.string ==~"(?i)C"}
{Token.string ==~"57"}
{Token.string ==~"(?i)bl"}
{Token.string =="/"}
{Token.string ==~"6"}
({Token})[0,1]
{Token.string ==~"(?i)mice"}
)
|
(
{Token.string==~"(?i)aged"}
((numbers)
({Token})[0,1]
(numbers)?
(weeks)
({Token})[0,1]):age
({Token.string==~"(?i)were"}|{Token.string==~"(?i)are"})
)
|
(
((numbers)
({Token})[0,1]
{Token.string==~"(?i)to"}
(numbers)
({Token})[0,1]
(numbers)?
(weeks)):age
(gender)
)
|
(
(gender)
({Token})[0,1]
((numbers)
({Token})[0,1]
(numbers)?
(weeks)):age
)
|
(
((numbers)
({Token})[0,1]
(numbers)?
(weeks)):age
(gender)
)
|
(
(wholte_string_age):age
({Token})[0,1]
(gender)
)
|
(
((wholte_string_age2)
({Token})
(numbers)
({Token})[0,1]
(numbers)?
(weeks)):age
(gender)
)
|
(
((whole_string_age2)
({Token})
(wholte_string_age)
({Token})[0,1]
{Token.string ==~"(?i)old"}):age
(gender)
)
|
(
{Token.string ==~"(?i)were"}
({Token.string ==~"(?i)purchased"}|{Token.string=~"(?i)used"})
({Token})[0,1]
((numbers)
({Token})[0,1]
(numbers)?
(weeks)):age
)
|
(
{Token.string==~"(?i)aged"}
{Token.string==~"(?i)to"}
((numbers)
({Token})[0,1]
(numbers)?
(weeks)):age

)
